# General hospital patients’ satisfaction with a proactive automatized multiple health behavior change intervention

**DOI:** 10.1186/s12913-025-13425-x

**Published:** 2025-09-23

**Authors:** Caroline Timm, Anika Tiede, Filipa Krolo-Wicovsky, Marie Spielmann, Gallus Bischof, Christian Meyer, Ulrich John, Jennis Freyer-Adam

**Affiliations:** 1 Institute for Community Medicine, Department of Prevention Research and Social Medicine, University Medicine Greifswald, Greifswald, Germany; 2 German Centre for Cardiovascular Research e.V, Partner site Greifswald, Greifswald, Germany; 3 Institute for Medical Psychology, University Medicine Greifswald, Greifswald, Germany; 4 Department of Psychiatry and Psychotherapy, University of Lübeck, Lübeck, Germany

**Keywords:** Multiple health behavior change, Behavior change intervention, Satisfaction, Digital feedback, Social equity

## Abstract

**Background:**

Behavior change interventions targeting multiple health risk behaviors are needed to reduce the burden of non-communicable diseases. This study investigates general hospital patients’ satisfaction with process and outcome of a proactive automatized lifestyle intervention and whether characteristics of the patients (i.e. age, sex, level of school education, number of health risk behaviors, presence of any non-communicable disease) are associated with intervention satisfaction.

**Methods:**

Between May and July 2022, all 18- to 64-year-old patients in five medical departments at the University Medicine Hospital Greifswald, Germany were systematically approached. The intervention included individualized computer-generated feedback letters regarding patients’ individual profile of health risk behaviors, and tailored to the current motivational stage of change. Feedback was scheduled to be delivered after baseline, month 1 and month 3. Of 175 intervention participants, 135 (77.1%) participated in the follow-up after 8 months, and reported on satisfaction with intervention process and outcome. Associations of overall satisfaction with patient characteristics were analyzed using linear regression analysis.

**Results:**

81% (*n* = 107) of all follow-up participants rated the entire intervention as good or very good; and 94.0% (*n* = 124) reported no experienced discomfort linked to the intervention participation. Mean satisfaction was 2.9 (SD = 0.8, range 0–4). Low level of school education (*p* = 0.02) was positively related to intervention satisfaction.

**Conclusions:**

General hospital patients, and those with low level of school in particular, were rather satisfied with a proactive multiple health behavior change intervention containing computer-generated stage-tailored written feedback. Findings highlight patients’ positive perceptions of proactively delivered and individually tailored feedback on their health risk behaviors; and support the implementation of proactive multiple health behavior change interventions as part of routine health care.

**Trail registration:**

ClinicalTrials.gov Identifier: NCT05365269 on May 9, 2022.

**Supplementary information:**

The online version contains supplementary material available at 10.1186/s12913-025-13425-x.

## Background

To reduce the burden of non-communicable disease caused by health risk behaviors (HRBs), suitable behavior change interventions are needed [1]. Major HRBs include tobacco smoking, at-risk alcohol use, unhealthy diet and physical inactivity. At least half of the adults in Germany and many other countries indicate two or more of these HRBs [2, 3]. This great proportion is particularly crucial as the co-occurrence of HRBs increases the risk for mortality more than additively [4, 5].

Multiple health behavior change interventions are characterized by addressing two or more health risk behaviors either simultaneously or sequentially within a limited time period [6]. Previous studies reported positive behavior change effects when targeting multiple HRBs in individuals with non-communicable diseases [7]. Primary care patients aged 45 to 75 who received a multiple health behavior change intervention on three target behaviors were more likely than controls to change two or all three targeted behaviors, e.g. reduced smoking, increased physical activity and/or adhered to the target diet [8].

Recent findings from an own study showed that more than 90% of systematically approached general hospital patients approved systematic health risk behavior screening and intervention at least somewhat, 58% highly [9]. Additionally, 82% agreed to participate in a proactive multiple health behavior change intervention [10]. Understanding the patient-centered perspective on the actual delivery of behavior change interventions requires further investigation into patients’ satisfaction with such interventions [11]. Current research predominantly emphasized the feasibility and efficacy of behavior change interventions [7, 12], which permitted only generalized assumptions about patient satisfaction and lead to a significant lack in research. Single studies revealed rather high satisfaction with multiple health behavior change interventions among a small and selective sample and derived from an uni-dimensional measurement of satisfaction [8, 13]. However, treatment satisfaction is a multi-dimensional construct including the participants’ appraisal of intervention attributes concerning process and outcome [14]. Process attributes include the suitability and utility of the intervention components, attitude toward and desire to continue with intervention, competence and interpersonal style of interventionist and format and dose of the intervention. Outcome attributes include improvement in the health problem and in every day functions, discomfort and attribution of the outcomes to the intervention [15].

Intervention satisfaction is associated with intervention adherence and outcome [14, 16], and intervention characteristics such as type of therapy or method of assignment to therapy have been found to be more strongly related to satisfaction than participant characteristics [17]. However, little is known with regards to whether patient characteristics are related to satisfaction with multiple health behavior change interventions targeting tobacco smoking, at-risk alcohol use, unhealthy diet and physical inactivity in the general hospital setting. Patient characteristics, such as school education in particular, are worthwhile to investigate given the major challenge of public health approaches to reduce social inequalities in health services which are to a large extend explained by differences in these HRBs and participation in health behavior change interventions [18–20].

The aim of this study was to investigate the satisfaction of general hospital patients with process and outcome of a proactively delivered automatized multiple health behavior change intervention as mentioned above [21]. Furthermore, we investigated whether beyond intervention characteristics, patient characteristics (i.e. level of school education, age, sex, total number of HRBs, presence of a non-communicable disease) are also related to satisfaction with the intervention.

## Methods

### Sampling frame and participants

Data from the pre-post-intervention study “Proactive automatized lifestyle intervention for cancer prevention: Pilot-test (PAL-Pilot)” were used [22]. The study was approved by the data protection officer and ethics committee of the University Medicine Greifswald (BB 024/17; BB 024/17a).

**Fig. 1:**
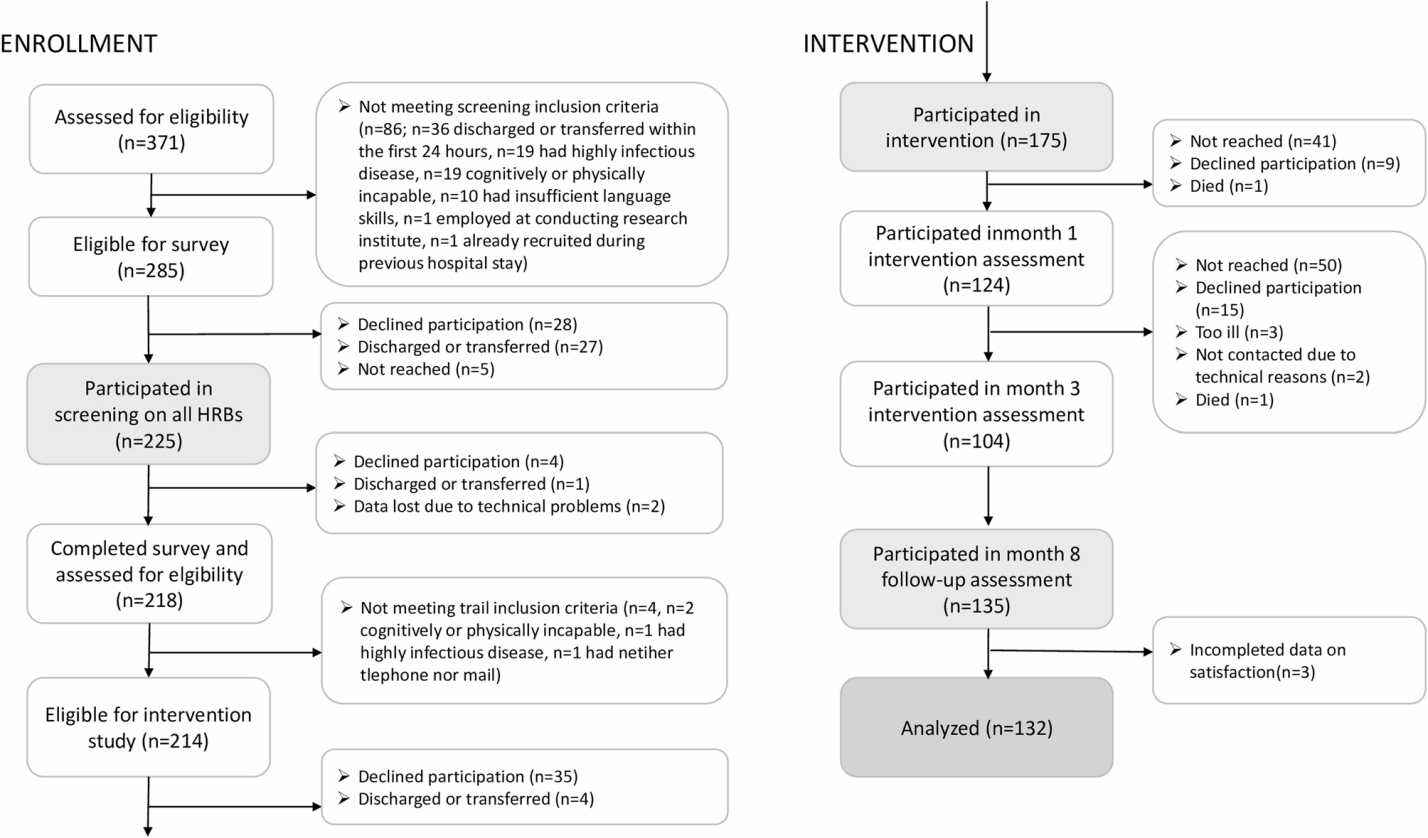
Patient flow for intervention and follow-up paticipation

Proactive recruitment took place between May and July 2022 at the University Medicine Hospital Greifswald in Germany on nine wards: general and thorax surgery, trauma surgery, otorhinolaryngology, gastroenterology, endocrinology, nephrology, cardiology, angiology, pneumology. On Tuesdays through Fridays, all patients aged 18–64 years of all sexes and admitted the day before were eligible for inclusion, were approached by a research assistant and were asked to fill in a screening on health behaviors using tablet computers. Patients were excluded if they met any of the following criteria: cognitive or physical incapacity, presence of a highly infectious disease, discharged or transferred outside the study area within the first 24 h of admission, insufficient language skills or employment at the conducting research institute. As described in Fig. 1 and reported in more detail elsewhere [10], 225 of all 285 eligible patients participated in the screening. Of those 214 who completed the screening and eligible for intervention, 175 (81.8%) participated in the intervention study and provided written informed consent.

### Intervention

The Proactive Automatized Lifestyle intervention (PAL) was delivered in the form of computer-generated letters after baseline, after month 1 and after month 3; and targeted tobacco smoking, at-risk alcohol use, diet and insufficient physical activity as described in more detail elsewhere [22]. Proactivity refers to high efforts used to reach all patients by individually approaching them and proactively contacting each participant at each time-point. Proactivity results in high reach, also of those people who are not yet ready to change and/or ready to actively search for such an intervention [1, 21]. High reach is promising in terms of public health impact [23], but also highly relevant for how interventions need to be designed [1]. Automatization refers to highly individualized feedback generated by an expert system software which contained a pool of more than 3000 text modules as well as supporting graphics. Based on assessment data and predefined allocation rules, the software selected appropriate text modules and graphics to individually tailor each feedback letter to the participants’ current stage of change. The theoretical basis, the transtheoretical model of intentional behavior change [24], was originally developed to explain and support behavior change, also among the vast majority of people not yet ready to change. It postulates that persons proceed through different stages of change, and that persons in different stages require different, i.e. stage-tailored, interventions.

First all participants received individualized information on which of the four HRBs were present and not present (See Additional File 1). The intervention then provided more detailed and motivation-enhancing feedback for those HRBs present. To avoid overwhelming patients with three or four HRBs, two HRBs at maximum were selected based on participants’ perception of which HRB change would have the greatest positive impact on their life and on evidence-based considerations as described elsewhere [25]. Furthermore, the intervention included public health recommendations as well as normative and ipsative feedback. Normative feedback reflected on the participant’s current data in comparison to comparable other persons with regards to gender or motivational stage. Ipsative feedback reflected on the participant’s own motivational and behavioral changes from the previous to the current timepoint.

For the two post-baseline interventions, all 175 participants were contacted by phone to conduct computer-assisted telephone interviews to collect assessment data, on which the feedback was based. Of these, 24.0% (*n* = 42), 21.7% (*n* = 38) and 54.3% (*n* = 95) received 1, 2 and 3 feedbacks by ordinary mail, respectively, with a mean intervention dose of 2.3 (SD = 0.8) [10]. As a consequence of the Covid-19 pandemic and technical issues, the originally scheduled month 3 feedbacks were delayed to month 4.

### Follow-ups

PAL participants were contacted at month 8 by email to fill in self-administered online questionnaires, originally scheduled for month 6. To enhance follow-up participation, prepaid incentives were sent out (i.e. previously self-selected 10 Euro-vouchers), up to three reminders were sent out by email, and in case of missing response, study staff contacted participants to conduct computer-assisted telephone interviews. If these contact options failed, a paper-pencil questionnaire was sent out by ordinary mail. Of all 175 intervention participants, 135 (77.1%) participated in the follow-up; *n* = 85 provided their data through participation in computer-assisted telephone interviews, *n* = 34 through online participation and *n* = 16 by paper-pencil.

### Measurements

Self-report was used (Additional file 2). To check whether the feedback letters had been read, participants were asked “How profoundly did you read the letters?” (not at all/partially/completely). Participants who answered “not at all”, were further asked “Did you keep the letters?” (yes/no). Participants who answered “partially”, were asked “Are there any letters that you have not read at all?” (yes/no). Participants who answered “completely”, were asked “Are there any letters that you have read several times?” (yes/no).

Satisfaction with intervention was measured asking the participants to grade the intervention from 1 (very good) to 6 (deficient) and by using the structure of the multi-dimensional treatment satisfaction measure [14] and items as assessed in Krause et al. [13]. The multidimensional measure incorporated 5-point Likert rating scales (0, strongly disagree to 4, strongly agree) and was computed as the mean of the respective items. Satisfaction with process was assessed using five subscales: suitability (3 items, e.g. feedback was comprehensible, Cronbach’s α = 0.81), utility (4 items, e.g. feedback was helpful, α = 0.87), attitude toward intervention (2 items, e.g. participation was worthwhile, α = 0.76), recommendation to other persons (1 item) and format and dose (3 items, e.g. the amount per feedback was appropriate, α = 0.85). Satisfaction with outcome was assessed using six items. One item assessed the overall attribution of outcomes to intervention (“The intervention encouraged me to make changes”). Four items assessed perceived benefits for each HRB (“Because of the study participation I intend to smoke less [or no] tobacco/drink less [or no] alcohol/pay more attention to healthy diet/be more physically active”, yes/no). One item assessed discomfort (“Have you experienced any discomfort or unpleasant side effects linked to the feedback?”, yes/no). If so, a free text field was provided to report any unpleasant experiences. Total satisfaction with intervention was measured by a total mean score (range: 0–4) computed of all five process subscales and the one item on attribution of outcomes to the intervention (α = 0.90).

Suggestions for improvements were assessed among participants who disagreed with format and dose (scores 0–2). Corresponding items were provided: “What amount per feedback/frequency of feedback/intervals between feedbacks would you consider appropriate?” with response options fewer/more and smaller/larger, respectively.

Patient characteristics were assessed at baseline. Socio-demographics included school education categorized into low (< 10 years of school), medium (10–11 years) and high level (> 11 years), age and sex (male/female). Behavioral HRBs were assessed individually and summed up (0–4). As described in more detail elsewhere [9], any current daily or occasional tobacco smoking was considered as HRB. At-risk alcohol use was determined using the Alcohol Use Disorder Identification Test-Consumption [26] and cut-off values of ≥ 4 for women and ≥ 5 for men [27], corresponding to the national guidelines [28]. Unhealthy diet was determined using the question “How many servings of vegetable and fruit do you eat on average per day?”. Less than 5 servings were considered as unhealthy diet [29]. Physical inactivity was assessed using an adapted version of the European Health Interview Survey-Physical Activity Questionnaire [30]. In accordance to recommendations of the World Health Organization [31], less than 150 min per week of moderate and/or less than 75 min per week of vigorous physical activity were considered as insufficient physical activity.

Any non-communicable disease was considered when cancer, a cardiovascular disease, a chronic respiratory disease or diabetes mellitus were reported. The four non-communicable diseases were assessed by one question each: “Have you ever been diagnosed by a doctor with [cancer/cardiovascular disease/chronic respiratory disease/diabetes mellitus]?”. Examples of diseases were provided for cardiovascular disease (hypertension, myocardial infarction, coronary heart disease, stenocardia, myocardial insufficiency, heart failure) and chronic respiratory disease (chronic bronchitis, chronic obstructive pulmonary disease, pulmonary emphysema). Of all six responses, those three indicating a diagnosis during the current hospital stay, within the past 12 months or more than 12 months ago were considered as the respective disease being present.

Intervention characteristics including information on intervention module (i.e. smoking, alcohol, diet, inactivity) and intervention dose (i.e. 1–3) delivered to participants were derived from the participant management documentation.

### Statistical analysis

To determine patients’ satisfaction with the intervention, the number of cases (N) and proportions (%) among eligible patients per item, the means and standard deviations (SD) of the subscales, and the total satisfaction score were provided. To investigate associations between patient characteristics and satisfaction, a multivariable linear regression was calculated with assorted and potentially relevant socio-demographic variables (i.e. age, sex, level of school education), number of HRBs (i.e. 1–4) and presence of any non-communicable disease (i.e. yes/no) as predictors, and controlled for relevant intervention characteristics (i.e. feedback modules received, and intervention dose).

To investigate whether the 135 follow-up participants differed from intervention participants who did not provide follow-up data concerning age, sex, level of school education, number of HRBs, and presence of any non-communicable disease and to adjust potential confounding, a multivariate logistic regression was conducted.

All cases with missing values were excluded listwise. Due to missing values in three intervention participants, the data of 132 follow-up participants was analyzed. Statistical significance was tested with *p* < 0.05 and the Stata program 17.0 was used for all data analyses [32].

## Results

### Sample characteristics

The 175 intervention participants are described in more detail elsewhere [10]. 135 follow-up participants of these were on average 51.7 years old (SD = 12.2), 51.9% (*n* = 70) were men, 68.2% (*n* = 92) had a medium level of school education, 53.3% (*n* = 72) had at least one non-communicable disease, 98.5% (*n* = 133) reported one or more of the four HRBs and the mean number of HRBs was 2.1 (SD = 1.0). They were significantly older (*p* = < 0.001), more often reported no non-communicable disease (*p* = 0.022), and were by trend more likely women (*p* = 0.073) than follow-up non-participants (Table 1).

**Table 1:** Baseline characteristics of follow-up sample (*n* = 135) in comparison to the follow-up non-participants (*n* = 40)

Variables	Follow-up participants		Follow-up non-participants		Odds Ratio	95% confidence interval	*p*
	*N*	%	*N*	%			
Socio-demographics							
Age (M, SD)	51.7	12.2	41.7	12.6	1.1	1.0–1.1	< 0.001
Sex							
Men (reference)	70	51.9	27	67.5			
Women	65	48.2	13	32.5	2.1	0.9–4.9	0.073
Level of school education							
Low (reference)	19	14.1	7	17.5			
Medium	92	68.2	24	60.0	1.0	0.3–2.9	0.942
High	24	17.8	9	22.5	1.6	0.4–6.0	0.519
Present behavioral health risk behaviors*							
Health risk behavior number (M, SD)	2.1	1.0	2.4	1.0	0.8	0.5–1.2	0.274
Tobacco smoking	42	31.1	19	47.5		not included	
At-risk alcohol use	37	27.4	66	29.3		not included	
Unhealthy diet	126	93.3	40	100.0		not included	
Physical inactivity	79	58.5	21	52.5		not included	
Present non-communicable diseases*							
Any	72	53.3	23	59.0	0.3	0.1–0–9	0.022
Cancer	20	14.8	2	5.1		not included	
Cardiovascular diseases	51	37.8	15	38.5		not included	
Chronic respiratory disease	19	14.1	4	10.3		not included	
Diabetes mellitus	23	17.0	7	18.0		not included	

M = Mean, SD = standard deviation, N = number of cases, *p* = *p*-value, *multiple responses possible, the table includes all variables entered in the regression

### Intervention check

Of all follow-up participants, 72.9% (*n* = 97) reported they had completely read the feedback letters; 25.8% (*n* = 25) of them several times. 22% (*n* = 29) reported they had partially read the feedback letters; 82.8% (*n* = 24) of them read all letters to some extent. 5% (*n* = 7) reported they had not read the feedback letters at all; one of them had kept the letters.

### Satisfaction with intervention

**Fig. 2:**
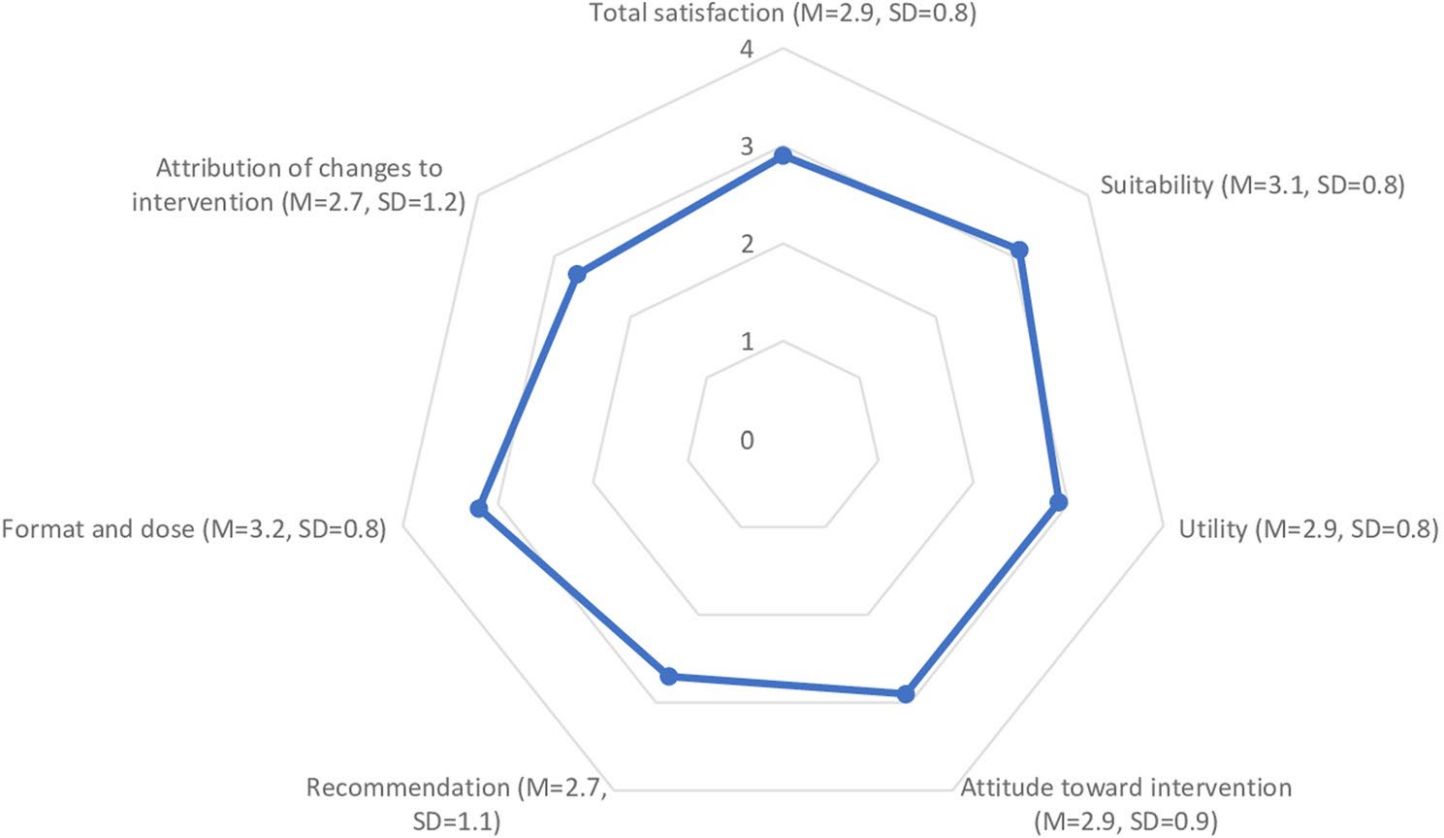
Satisfaction with intervention

Of all follow-up participants, 80.5% (*n* = 107) graded the intervention as good (60.9%, *n* = 81) or very good (19.6%, *n* = 26). The mean overall score of satisfaction with intervention was 2.9 (SD = 0.8, Table 2). Satisfaction with process ranged between 2.7 (SD = 1.1) for recommendation of intervention to others and 3.2 (SD = 0.8) for dose and format. Satisfaction with outcome as indicated by attribution of intentional changes in HRBs to intervention was 2.7 (SD = 1.2, Fig. 2).

**Table 2:** Satisfaction with a proactive automatized multiple health behavior change intervention on item level

Domain	Subscale	Item	*N*	M	SD	Observed range
Process	Suitability	Feedback was comprehensible	133	3.3	0.8	0–4
		Feedback was tailored to my personal situation	132	2.9	1.0	0–4
		Feedback was presented attractively	133	3.1	0.8	0–4
	Utility	Feedback was helpful	133	2.8	1.0	0–4
		Feedback was motivating	133	2.6	1.0	0–4
		Feedback was interesting	133	3.0	0.8	0–4
		Feedback was respectful	133	3.2	0.8	0–4
	Attitude toward intervention	Expectations were fulfilled	133	3.0	0.9	0–4
		Participation was worthwhile	133	2.8	1.0	0–4
	Recommendation	Recommendation to other persons	133	2.7	1.1	0–4
	Format and dose	Amount of feedback was appropriate	133	3.2	0.9	0–4
		Frequency of feedback was appropriate	133	3.2	0.9	0–4
		Intervals between feedbacks were appropriate	133	3.3	0.9	0–4
Outcome	Attribution of changes in behavior change intention to intervention	Intervention encouraged me to make differences	133	2.7	1.2	0–4
Total satisfaction			132	2.9	0.8	0–4

*N* number of cases, *M* mean, *SD* standard deviation

Attribution of intentional changes in HRBs to study participation was high. Specifically, 46.0% (17/37) of the participants with at-risk alcohol use, 53.7% (22/41) of those who smoked tobacco; 78.4% (98/125) of those with unhealthy diet, and 80.8% (63/78) of those with insufficient physical activity at baseline reported that because of study participation, they tried to drink less (or no) alcohol/to smoke less (or no) tobacco/to eat healthier or to be physically more active, respectively. 94% (*n* = 124) reported no experienced discomfort or unpleasant side effects linked to the intervention. Of those nine participants who did, five reported dissatisfaction with the amount and frequency of the intervention (2.9% of 175), three reported discomfort with the focus on substance-related behavioral HRBs (1.7% of 175); and one reported that negative family memories came up through intervention participation (0.6% of 175).

### Participants suggestions for improvement

Among the 22 participants not satisfied with the amount of feedback, 18 (10.3% of 175) suggested less and 4 (2.3% of 175) more feedback per letter. Among the 15 participants not satisfied with the frequency of feedback letters, 11 (6.3% of 175) suggested to receive fewer and 4 (2.3% of 175) more letters. Of the 16 participants not satisfied with the intervals between feedback letters, 11 (6.3% of 175) suggested smaller and 5 (2.9% of 175) larger intervals.

### Associations of patient characteristics with overall satisfaction

Among all patient characteristics, medium versus low level of school education (β=−0.4; *p* = 0.02) was negatively related to overall satisfaction. Both covariates were positively related to total satisfaction, namely the mean dose of intervention (β = 0.4; *p* = < 0.001) and having received the module diet (β = 0.5; *p* = 0.02, Table 3).

**Table 3:** Patient and intervention characteristics in relation to intervention satisfaction

	β	*p*
Patient characteristics		
Socio-demographics		
Age in years	0.01	0.063
Women versus men	0.2	0.123
Medium versus low level of school education	−0.4	0.022
High versus low level of school education	−0.3	0.186
Mean number of health risk behaviors	−0.1	0.639
Any non-communicable diseases (yes versus no)	−0.04	0.772
Intervention characteristics		
Mean dose of intervention	0.4	< 0.001
Intervention module received		
Smoking (yes versus no)	0.3	0.401
Alcohol (yes versus no)	0.2	0.402
Diet (yes versus no)	0.5	0.021
Inactivity (yes versus no)	0.3	0.158

Multivariable linear regression, *n* = 132, β = coefficient; *p* = *p*-value, the table includes all variables entered in the regression

## Discussion

This study revealed two key findings. First, 81% of general hospital patients who were followed-up were quite or very satisfied with a proactive automatized intervention to foster healthy behaviors. Second, low level of school education compared to medium level of school education was positively associated with satisfaction.

Our findings underline high patient satisfaction with a proactively delivered and automatized multiple health behavior change intervention and support the implementation of health promoting interventions in health care settings, also from the patient perspective [8, 13]. Considering the multidimensional nature of intervention satisfaction [14], our data revealed that participants were overall satisfied with both, intervention process and outcome. In line with findings from other brief intervention studies [8], experienced discomfort linked to participation in the behavior change intervention was rather tolerable. Most of the small proportion of participants not so satisfied with intervention dose (9%) suggested reduced feedback per letter, fewer letters and smaller intervals between letters. This speaks in favor of the assumption that participants prefer compressed interventions. With respect to our intervention, delivering the last intervention at month 3 as per protocol instead at month 4 might have been perceived as more appropriate. Although only one participant reported the resurfacing of negative family memories during the intervention, it appears helpful to be aware of potential emotional reactions, and to include information on the access to psychological support throughout and after the intervention as part of future implementations of such interventions, e.g. as part of the informed consent procedure.

In line with previous findings [17], most of the patient characteristics were not related to satisfaction with intervention. However, our study revealed that patients with a low level of school education reported greater satisfaction with the behavior change intervention than participants with a medium level of school education. Given that, patients with a low level of school education are particularly affected by multiple HRBs [33], their particular satisfaction may be explained by appreciating the delivery of individualized feedback and information in a patient-supportive and motivation-enhancing manner. Together with previous findings of the same study showing that intervention reach and retention were independent of the level of school education [34], our new findings indicate that proactive computer-based multiple health behavior change interventions may be acceptable by the vast majority of general hospital patients including those most in need. Given that persons with low school education are often hard to reach [20], this finding seems particularly promising in terms of social equity impact and prevention efforts to reduce social inequalities in health services [19]. Given that ten patients were excluded from our study due to insufficient language skills, future interventions should aim to further improve inclusiveness by providing assessments and feedback in multiple languages. This may be increasingly easily done in the future, and represents a particular strength of electronic interventions in better addressing social inequalities in health.

In line with previous findings, intervention adherence was positively associated with intervention satisfaction [16]. Higher satisfaction with the content, delivery, or perceived relevance of the intervention may enhance participants’ motivation to continue engaging with it. When individuals find an intervention valuable and tailored to their needs, they are more likely to remain committed and complete subsequent components. Conversely, lower satisfaction may lead to drop-out or reduced participation over time. The greater satisfaction with the diet module may be explained by greater openness of patients towards energy-balance related HRBs [35], greater stigmatization of substance use related HRBs such as tobacco smoking and at-risk alcohol drinking [36], and insufficient power for the modules providing feedback for the less common HRBs. The diet module was offered to 105 participants in our study while only 26 to 61 participants received the modules alcohol, tobacco smoking and physical activity, respectively.

### Limitations and strengths

The following strengths and limitations should be noted when interpreting the study findings. Strengths include the application of a multi-dimensional assessment of intervention satisfaction [14] and the satisfactory participation rates. The majority of patients participated in each phase of the study. That is, 79% of all eligible patients participated in the initial survey, 82% of those approached and eligible patients participated in the intervention and 77% completed the follow-up survey. Limitations include: First, although self-report is a well-established data source for the valid assessment of people’s perceptions and for implementing behavior change interventions, some patient responses assessed as potential predictors of satisfaction with intervention (e.g. on HRBs) may be biased by social desirability. However, in this study potential self-report bias may be reduced as investigated variables were not in the focus of the intervention and as validated measures like the Alcohol Use Disorders Identification Test-Consumption [27] were used whenever possible. Second, different from the original measure, our total satisfaction score contained one outcome item only. Instead, additional separate descriptive measures provided further information, e.g. on unwanted side effects. Third, some differences may not have been found significant due to the small subsample sizes as described above. Fourth, various data collection methods were employed during the follow-up assessments, including online questionnaires, paper-pencil surveys, and computer-assisted telephone interviews. While this approach helps to increase response rates, it may introduce method-related biases. To minimize bias, all research staff received thorough training to ensure consistent and uniform administration across methods. Fifth, satisfaction with the intervention remains unknown for 23% of the initial intervention participants. To statistically control our analyses for any patient characteristics that were significantly or by trend related to drop-out, our analyses were adjusted for age, gender and any non-communicable disease.

## Conclusion

From the patient perspective, the implementation of multiple health behavior change interventions to facilitate health risk behavior changes while hospitalized and beyond is appreciated. Concerning future intervention development, patients seem to appreciate compact feedback driven by psychological behavior change theory, tailored to ones own current motivational stage of change and needs, and provided in a patient-supportive and non-confrontational style. What is more, our findings indicate that proactive delivery and supportive feedback may be suitable intervention components to tackle social inequality in health, a core challenge in public health [18, 19], as the intervention was particularly valued by patients with low school education. These information are particularly valuable in terms of social equity impact of interventions as they provide potential solutions to successfully address this population group which is most in need given the great accumulation of HRBs [2, 3] and which is particularly hard to reach for prevention efforts [20].

## Supplementary Information

Below is the link to the electronic supplementary material.

Supplementary Material 1

Supplementary Material 2

## Data Availability

The dataset analyzed during the current study is not publicly available due to the German data protection law but are available from the principle investigator of the study, Prof. Dr. Jennis Freyer-Adam on reasonable request that complies with the study purpose and the participants` informed consent.

## Abbreviations

β: beta coefficient
HRBs: health risk behaviors
M: mean
n: number of cases
PAL-Pilot: Proactive automatized lifestyle intervention for cancer prevention: Pilot-test
*p*: *p*-value
SD: standard deviation
