# Supplementary material for: General hospital patients’ satisfaction with a proactive automatized multiple health behavior change intervention
Source: BMC Health Serv Res. 2025 Sep 23;25:1209. doi: 10.1186/s12913-025-13425-x (PMC12459032; doi:10.1186/s12913-025-13425-x)
Supplement: Supplementary file 1 — Supplementary Material 1 [file 12913_2025_13425_MOESM1_ESM.pdf]

Supplement of the manuscript

Caroline Timm, Anika Tiede, Filipa Krolo-Wicovsky, Marie Spielmann, Gallus Bischof, Christian Meyer, Ulrich John, Jennis Freyer-Adam **"General hospital patients' satisfaction with a proactive automatized multiple health behavior change intervention" submitted for publication in BMC Health Services Research**

This supplement contains further information on the feedback letters generated as part of the Proactive Automatized Lifestyle intervention (PAL) and two examples:

1) Supplement Table 1: Feedback content of computer-generated letters for each intervention module in the order of appearance.
2) Example letter 1: This month 0 feedback letter addresses a female patient with insufficient vegetable and fruit intake which she is ready to increase and with at-risk alcohol use which she is currently not intending to change. The differently colored numbers refer to the content in Supplement Table 1.
3) Example letter 2: This month 3 feedback letter addresses a male patient with all four health risk behaviors and who has not improved any of these behaviors since the last assessment, and for whom physical activity and tobacco smoking were selected after baseline for more detailed motivation-enhancing feedback. He is ambivalent to change this to health risk behaviors. The differently colored numbers refer to the content in Supplement Table 1.

Supplement Table 1: Feedback content of computer-generated letters for each module in the order of appearance

Order	Number of pages	Module and respective content
1	1	**MODULE PROFILE**
		**1. Opening.** Letter head University Medicine Greifswald, address, personalized greeting "Dear Mrs … / Dear Mr …"
		**2. Expressing gratitude** for study participation and information on now receiving feedback on physical activity, diet, alcohol use, tobacco smoking.<br>Additions at months 1 & 3: Information on receiving feedback on own changes since hospital stay / last feedback.
		**3. Visualizing individual health risk behavior profile:**<br>Green symbols indicate recommendations are met; orange symbols indicate potential areas of potential improvement.<br>Brief description of graphic and information on which topics are selected for the following more detailed feedback.<br>Additions at months 1 & 3: The symbol "sun" indicates significant behavioral improvements since the last feedback.
		**4. Personalized closure** "Mrs … / Mr… all the best! With kind regards, … name of principle investigator"
2*	2-2.5	**MODULE PHYSICAL ACTIVITY**
		**1. Emphasizing autonomy:** "In case you are interested: You are now receiving your (new) feedback on your physical activity based on data provided by yourself".
		**2. Where are you now?** Feedback on current stage of behavior change, number of minutes spent with physical activity per week; and on own muscle strengthening activities.<br>Month 0: Behavioral feedback is provided in comparison to other women/ men (normative comparison), supported by a graph.<br>Months 1 and 3: Feedback ion on behavioral or stage changes/ non-changes since last feedback (ipsative comparison).
		**3. Visualization of WHO recommendations**, accompanied by descriptions of moderate and vigorous activity and examples.
		**4. Feedback on decisional balance**, i.e. on own perceived pros and cons of changing, also in comparison to others in the same stage<br>Raising open questions to reflect for oneself<br>Months 1 and 3: Feedback on own changes since last feedback.
		**5. How can you proceed on?** Feedback on process use, also in comparison to others in the same stage<br>Participants not yet ready to change (precontemplation, contemplation) receive feedback on four cognitive-experiential processes<br>Participants ready to change (preparation, action) receive feedback on four behavioral processes<br>Additions at months 1 & 3: Feedback on own changes since last feedback.
		**6. Beware of difficult situations.** Feedback on self-efficacy in comparison to others in the same stage.<br>Only for participants in contemplation and later stages.<br>Raising open questions to reflect on more active times, own strengths and own achievements.<br>Presenting a range of strategies to pick from for individual difficult situations that could help to handle these successfully.
		**7. When-how-where to change.** Encouraging the development of an own change plan supported by different examples of others, goal-oriented targeted open questions and visualization.<br>Only for participants in preparation and action stage.
		**8. Keep going!** Appreciation of achievements, pointing out to keep letter in case it may be of interest at a later time point.

2/ 3*	2-2.5	**MODULE DIET**<br>1. **Emphasizing autonomy:** "In case you are interested: You are now receiving your (new) feedback on your diet based on data provided by yourself".<br>2. **Where are you now?** Feedback on current stage of behavior change, and on own vegetable/fruit intake per day.<br>Behavioral feedback is supported by visualization<br>Additions at months 1 and 3: Feedback on behavioral or stage changes/ non-changes since last feedback (ipsative comparison).<br>3. **Description of recommendations concerning intake of vegetable and fruit intake** and brief information on disease prevention<br>4. **Feedback on own fiber, fat, salt and sugar intake** supported by visualization.<br>Green symbols indicate national/ WHO recommendations are met; and are encouraged by appreciation<br>Orange symbols indicate national recommendations are not met; and different ideas of how to reduce these are individually provided.<br>Autonomy is emphasized.<br>Additions at months 1 and 3: Feedback on own changes since last feedback.<br>5. **Feedback on decisional balance,** i.e. on own perceived pros and cons of changing, also in comparison to others in the same stage<br>Raising open questions to reflect for oneself<br>Additions at months 1 and 3: Feedback on own changes since last feedback.<br>6. **What now?** Feedback on process use, also in comparison to others in the same stage<br>Participants not yet ready to change (precontemplation, contemplation) receive feedback on four cognitive-experiential processes<br>Participants ready to change (preparation, action) receive feedback on four behavioral processes<br>Additions at months 1 & 3: Feedback on own changes since last feedback.<br>7. **Beware of difficult situations.** Feedback on self-efficacy in comparison to others in the same stage.<br>Only for participants in contemplation and later stages.<br>Raising open questions to reflect on more active times, own strengths and own achievements.<br>Presenting a range of strategies to pick from for individual difficult situations that could help to handle these successfully.<br>8. **When-how-where to change.** Encouraging the development of an own change plan supported by different examples of others, goal-oriented targeted open questions and visualization.<br>Only for participants in preparation and action stage.<br>9. **Keep going!** Appreciation of achievements, pointing out to keep letter in case it may be of interest at a later time point.
2/ 3*	2-2.5	**MODULE ALCOHOL**<br>1. **Emphasizing autonomy:** "In case you are interested: You are now receiving your (new) feedback on your alcohol use based on data provided by yourself".<br>2. **Where are you now?** Feedback on current stage of behavior change and on current alcohol use in comparison to other women/men. Feedback on alcohol use per week and on risk for any adverse consequences for this amount of alcohol use (both supported by individualized graphs) and on occasional heavy drinking.<br>Additions at months 1 and 3: Feedback on stage and behavioral changes/ non-changes since last feedback (ipsative comparison). Up to two graphs per participant supported visualization of change in weekly alcohol use and risk measures.<br>3. **Visualization of national recommendations**, accompanied by a description of standard sizes of alcoholic drinks.<br>Reference to achieving the optimum by abstaining from alcohol use, particularly in case of diseases.<br>4. **Feedback on decisional balance**, i.e. on own perceived pros and cons of changing, also in comparison to others in the same stage<br>Raising open questions to reflect for oneself

		Additions at months 1 and 3: Feedback on own changes since last feedback.<br>5. **How can you proceed on?** Feedback on process use, also in comparison to others in the same stage.<br>Participants not yet ready to change (precontemplation, contemplation) receive feedback on four cognitive-experiential processes<br>Participants ready to change (preparation, action) receive feedback on four behavioral processes<br>Additions at months 1 and 3: Feedback on own changes since last feedback.<br>6. **Beware of difficult situations.** Feedback on self-efficacy in comparison to others in the same stage.<br>Only for participants in contemplation and later stages.<br>Raising open questions to reflect on more active times, own strengths and own achievements.<br>Presenting a range of strategies to pick from for individual difficult situations that could help to handle these successfully.<br>7. **When-how-where to change.** Encouraging the development of an own change plan supported by different examples of others, goal-oriented targeted open questions and visualization.<br>Month 0: Only for participants ready to change (in preparation).<br>Additions at months 1 and 3: Only for participants ready to change or already changing (preparation or action stage).<br>8. **Keep going!** Appreciation of achievements, pointing out to keep letter in case it may be of interest at a later time point.
2/ 3*	2-2.5	**MODULE TOBACCO SMOKING**<br>1. **Emphasizing autonomy:** "In case you are interested: You are now receiving your (new) feedback on your smoking behavior based on data provided by yourself."<br>2. **Where are you now?** Feedback on current stage of behavior change and on current smoking behavior in comparison to other men / women in the same age range, supported by individualized graphs (Only for month 0).<br>Additions at months 1 and 3: Feedback on stage changes / non-changes since last feedback (ipsative comparison).<br>3. **Smoking or no smoking?** Feedback on decisional balance, i.e. on own perceived pros and cons of changing.<br>Asking participants in precontemplation and contemplation (Months 1 and 3 only contemplation) to make a list of cons for smoking.<br>Raising open questions to reflect for oneself.<br>For participants over 60 years of age: Information that smoking cessation will lead to better physical health and enhanced quality of life.<br>Additions at months 1 and 3: Feedback on own changes since last feedback; encouraging participants in preparation to make a list of pros and cons for quitting and for smoking.<br>4. **Beware of difficult situations.** Feedback on self-efficacy and strategies to help with handling difficult situations.<br>Month 0: Only for participants in contemplation and later stages.<br>Additions at months 1 and 3: Presenting a range of strategies to pick from for individual difficult situations that could help to handle these successfully.<br>5. **How can you proceed on?** Feedback on process use, also in comparison to others in the same stage.<br>Participants not yet ready to change (precontemplation, contemplation) receive feedback on four cognitive-experiential processes<br>Participants ready to change (preparation, action) receive feedback on four behavioral processes<br>Additions at months 1 and 3: Feedback on own changes since last feedback.<br>6. **Keep going!** Appreciation of achievements, pointing out to keep letter in case it may be of interest at a later time point.

Notes: * if applicable and selected.

Universitätsmedizin Greifswald / Institut für Medizinische Psychologie

Frau
Berta Beispiel
1 Beispielweg 1
11111 Beispielstadt

6. Juli 2022

Ihre persönliche Rückmeldung - "Ein Weg gesund zu leben"

Sehr geehrte Frau Beispiel ,

2 vielen Dank für Ihre Teilnahme an der Befragung zu Ihrem Lebensstil im Krankenhaus. Falls Sie interessiert sind, erhalten Sie nun Ihre persönliche Rückmeldung zu den Themen körperliche Aktivität, Ernährung, Alkoholkonsum und Tabakrauchen.

So können Erkrankungen vorgebeugt werden und hier stehen Sie.

3 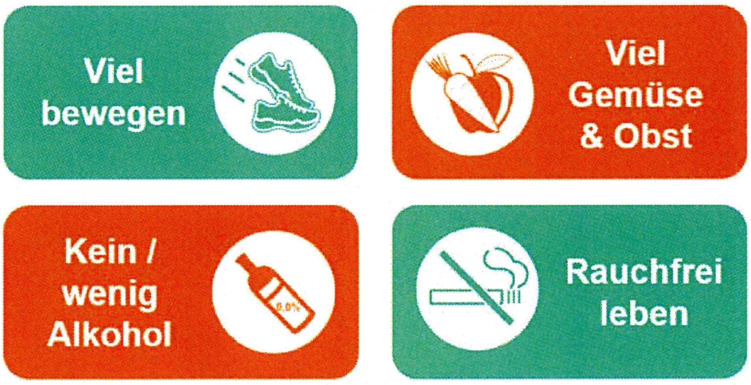

Sie sind körperlich aktiv und rauchen nicht. Das ist wirklich gut. Wenn Sie wissen möchten, wie Sie persönlich noch zusätzlich Erkrankungen vorbeugen können, dann schauen sie auf die orangen Symbole. In Ihrem Fall helfen Ihnen vielleicht die folgenden Rückmeldungen zu Ihrer Ernährung und zum Alkoholkonsum.

4 Wir wünschen Ihnen, sehr geehrte Frau Beispiel , alles Gute und viel Erfolg!
Mit freundlichen Grüßen

Freyer-Adam

Ihre Professorin Dr. J. Freyer-Adam

1 **Falls es Sie interessiert, erhalten Sie nun eine persönliche Rückmeldung zu Ihrer Ernährung, die sich aus Ihren Angaben ergibt.**

Wo stehen Sie?

2 Sie sind fest entschlossen, mehr Gemüse und Obst zu essen. Sehr gut! Damit haben Sie den nächsten Schritt zu einer gesunden Lebensführung gemacht.

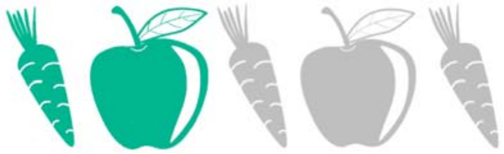

Von den empfohlenen 5 Portionen Gemüse oder Obst, essen Sie täglich 2 Portionen. Das ist ein guter Anfang.

Täglich 5 Handvoll Gemüse oder Obst...

3 … sind ein sehr guter Anfang für eine gesündere Ernährung. Empfehlenswert ist hierbei, dass unter den 5 Portionen mindestens 3 aus Gemüse bestehen. Diese können helfen weniger Zucker, Fett und Salz und ausreichend Ballaststoffe aufzunehmen. So können verbreitete Erkrankungen wie Krebs, Erkrankungen des Herz-Kreislaufsystems und Diabetes, sowie Übergewicht vorgebeugt werden.

Basierend auf Ihren Angaben sehen Sie an den orangen Symbolen, in welchen Bereichen Sie noch Veränderungen vornehmen können. Die grünen Symbole zeigen Ihnen, in welchen Bereichen Sie sich schon gesund ernähren.

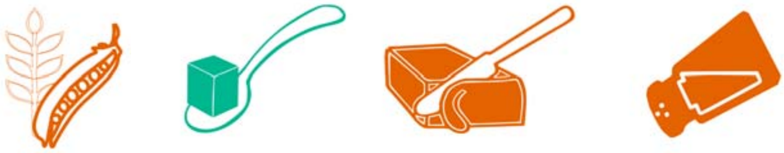

4 Sie nehmen wenig Zucker zu sich. Das ist gut!

- Die Aufnahme von Ballaststoffen kann z. B. durch einen höheren Verzehr von Vollkornbrot, Haferflocken, Nüssen, Gemüse und Obst gesteigert werden.
- Die Fettaufnahme kann z. B. durch einen geringeren Verzehr von Butter, Margarine, Fertigprodukten, Fleisch, Wurst, Käse, Eiern, vollfetten Milchprodukten, Salzgebäck, Süßspeisen, Schokolade und Backwaren gesenkt werden. Auf den Verzehr von fettreichem Fisch wie Hering oder Lachs sollte hingegen aufgrund seiner gesundheitsfördernden Wirkung nicht verzichtet werden.
- Die Salzaufnahme kann z. B. durch einen geringeren Verzehr von Fertigprodukten, Fleisch, Wurst, Käse, Brot oder Salzgebäck gesenkt werden.

Vielleicht ist etwas für Sie dabei?

5 **Für Sie überwiegen die angenehmen Seiten von Gemüse- und Obstverzehr.**

Das finden Sie nicht gut an Gemüse- und Obstverzehr: Sie fühlen sich danach oft aufgebläht.

Gut, dass Sie die folgenden Vorteile des Gemüse- und Obstverzehrs wahrnehmen: Gemüse und Obst

- verbessern Ihre Gesundheit
- Ihre Haut und Augen gesund zu halten
- erhöhen Ihre Energie

Im Vergleich zu anderen Menschen…

… erscheinen Ihnen die angenehmen Seiten des Gemüse- und Obstverzehr bedeutsam. Der nächste Schritt zu einem vermehrten Gemüse- und Obstverzehr ist nicht weit!

6 **Wie kommen Sie weiter?**

Oft hilft es einige Vorbereitungen zu treffen, um regelmäßig Gemüse und Obst in Ihren Speiseplan zu integrieren. Die folgenden Empfehlungen haben sich in vielen Fällen bewährt. Entscheiden Sie selbst, ob geeignete Anregungen für Sie dabei sind.

▶ *Haben Sie Vertrauen in sich selbst.*

Verglichen mit anderen, versprechen Sie sich selbst, dass Sie regelmäßig mehr Gemüse und Obst verzehren werden. Sehr gut! Diese Einstellung hilft Ihnen, Ihr Ziel zu erreichen.

▶ *Versuchen Sie sich zu überwinden.*

Auch wenn Sie ein weniger gesundes Lebensmittel bevorzugen würden, ziehen Sie Gemüse und Obst vor. Sehr gut! Das ist schon ein wichtiger Schritt dahin, regelmäßig Gemüse und Obst zu verzehren.

▶ *Bereiten Sie sich vor.*

Im Vergleich zu anderen nutzen Sie Strategien wie zur Hauptmahlzeit daran zu denken, Gemüse und Obst zu verzehren. Das ist sehr gut! Sich immer wieder Gemüse- und Obstverzehr ins Gedächtnis zu rufen, hilft Ihnen dabei, Ihr Ziel zu erreichen.

7 **Achtung: Zu überwindende Situationen**

Wenn die Hürden groß scheinen, fällt es einem oft nicht leicht, trotzdem Gemüse und Obst beim Essen zu integrieren. Gewohnheiten zu ändern ist dann besonders schwer. Es ist daher wichtig, die eigenen schwierigen Situationen zu kennen und richtig darauf zu reagieren.

Sie haben ein vergleichsweise hohes Zutrauen in Ihre Fähigkeit, auch in schwierigen Situationen bei Ihren Zielen zu bleiben. Sehr gut, bleiben Sie dabei! Das kann Sie auf Ihrem Weg zu einem gesundheitsbewussteren Lebensstil unterstützen.

Ihnen fällt es besonders schwer auf Gemüse und Obst in Ihren Mahlzeiten zu achten, wenn Sie gerade auswärts essen und eine schöne Zeit haben; nicht genug Zeit haben, mit Gemüse und Obst zu kochen und das Gemüse und Obst erst noch schälen und vorbereiten müssen. Folgendes kann hilfreich sein:

- Beim Restaurantbesuch auf Gemüse in den Beilagen achten
- Im Laufe des Tages die Gestaltung der Mahlzeiten genau planen
- Ihre wöchentlichen Mahlzeiten durchplanen und einen großen Wocheneinkauf täten
- Das Gemüse und Obst schon vorher schälen und vorbereiten
- Rezepte und Mahlzeiten auswählen, die für Sie leicht umsetzbar sind

8 Erstellen Sie einen Plan, wie Sie Ihre Vorsätze bezüglich vermehrtem Gemüse- und Obstkonsum einhalten können.

Um zukünftig regelmäßig Gemüse und Obst zu verzehren, ist es hilfreich, einen Plan zu erstellen. Denken Sie dabei an Ihre kritischen Situationen. Überlegen Sie: WIE, WO und WANN Sie Ihre Vorhaben bezüglich Gemüse- und Obstverzehr einhalten wollen. Je konkreter dieser Plan ist, desto leichter wird es, ihn im Alltag umzusetzen. Machen Sie es sich am Anfang nicht so schwer, setzen Sie sich realistische Ziele!

Beispielsweise wie Frau A., die sich vornimmt, jeden Tag Gemüse- und Obstportionen in Ihre Mahlzeiten einzubauen. Sie beschließt, ihr Frühstück mit einer Portion Obst und zum Mittag- und Abendessen jeweils die Beilagen mit Gemüse zu ergänzen (WIE). Damit möchte sie ab Montag (WANN) beginnen und bereitet dementsprechend alles in ihrem Kühlschrank vor (WO).

Wie könnte solch ein Plan zu mehr Gemüse- und Obstverzehr für Sie aussehen? Füllen Sie einfach die vorgegeben Zeilen aus und nehmen Sie sich fest vor, sich an Ihren Plan zu halten:

WANN? ____

WO? ____

WIE? ____

9 Bleiben Sie am Ball!

Auf dem Weg zu mehr Gemüse- und Obstverzehr haben Sie bereits eine entscheidende Hürde genommen: Sie planen ganz konkret Gemüse und Obst in Ihre Mahlzeiten einzubauen. Sehr gut! Wir hoffen, Ihnen interessante Hinweise gegeben zu haben, die Sie für Ihre Gesundheit und Ihr Wohlbefinden nutzen können. Bewahren Sie den Brief so auf, dass Sie ihn bei Bedarf noch einmal lesen können. Das bestärkt Sie darin, Ihren Plan in die Tat umzusetzen.

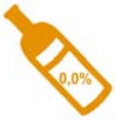

1 **Falls es Sie interessiert, erhalten Sie nun eine persönliche Rückmeldung zu Ihrem Alkoholkonsum, die sich aus Ihren Angaben ergibt.**

Wo stehen Sie?

2 Sie sehen derzeit keinen Grund über eine Änderung Ihres Alkoholkonsums nachzudenken. Über kleinere Bedenken grübeln Sie nicht weiter nach. Sie sind zufrieden, wie es ist. Wir möchten Ihnen hiermit einige Anregungen und Informationen geben.

Ein Vergleich Ihrer Trinkmenge mit der anderer

Wenn Sie wissen möchten, wie viel Sie im Vergleich zu anderen Frauen trinken, können Sie dies der Abbildung entnehmen. Ihr Alkoholkonsum pro Woche liegt innerhalb des orangenen Tortenstücks.

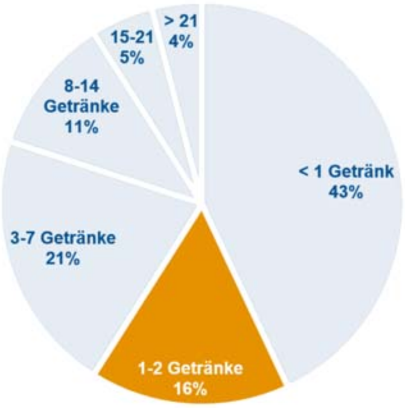

Wie 16% der Frauen im Alter von 18 bis 64 Jahren, trinken Sie 1 bis 2 alkoholische Getränke in der Woche.

Damit liegen Sie unter der empfohlenen Höchstmenge pro Woche. Prima!

3 Sie trinken nicht mehr als 7 kleine alkoholische Getränke pro Woche. Das ist gut so. **Aber:** Des öfteren trinken Sie mehr als 3 kleine alkoholische Getränke zu einer Gelegenheit. Damit haben Sie ein erhöhtes Risiko für negative Folgen des Alkoholkonsums. Sie können Ihr Risiko senken, indem Sie zukünftig höchstens 3 kleine Getränke zu einer Gelegenheit trinken.

JEDES Getränk WENIGER zählt!

Gesunde Frauen sollten:

- insgesamt **nicht mehr als 7** Getränke pro Woche
- und dabei **nicht mehr als 3** Getränke zu einer Gelegenheit

trinken.

Ein Getränk bedeutet:

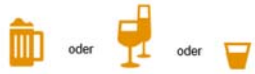

0,25-0,3l oder 0,1-0,15l oder 4 cl

Das heißt, wenn Frauen 3 mal in der Woche Alkohol trinken, sollten sie höchstens 2 kleine Getränke am Tag trinken. Jedes Getränk weniger oder kein Alkoholkonsum wäre optimal. Vor allem dann, wenn Erkrankungen vorliegen oder bestimmte Medikamente eingenommen werden.

4 Für Sie überwiegen die unangenehmen Seiten des Alkoholtrinkens.

Das finden Sie gut an Alkoholkonsum: Durch Alkohol entspannen Sie sich.

Gut ist, dass Sie den folgenden Nachteil in Ihre Entscheidung, Alkohol zu konsumieren oder nicht, einfließen lassen: Durch Alkohol

- schaden Sie Ihrer Gesundheit

Im Vergleich zu anderen Menschen ...

… finden Sie die unangenehmen Seiten nicht so bedeutsam. Es kann hilfreich sein, sie von Zeit zu Zeit neu zu durchdenken. So zeigt die Forschung, dass wir unserer Gesundheit etwas Gutes tun, wenn wir die Grenzen risikoarmen Konsums einhalten. Vielleicht sehen Sie auch ganz andere Vorteile des risikoarmen Trinkens? Achten Sie einmal darauf!

Oft ist es interessant zu überlegen, welche der angenehmen oder unangenehmen Seiten Ihnen momentan am wichtigsten sind. Danach könnten Sie überlegen, welche Ihnen langfristig mehr Vorteile bringen könnten.

5 Und was nun?

Die folgenden Empfehlungen wurden aufgrund Ihrer Angaben ausgewählt.

▶ *Überdenken Sie die Folgen.*

Sie sind häufiger betroffen, wenn Sie von negativen Folgen des Alkoholtrinkens hören. Dies hilft Ihnen, Vor- und Nachteile des risikoarmen Konsums einordnen zu können.

▶ *Achten Sie auf negative Folgen für Ihr Umfeld.*

Sie sehen zunehmend, dass das Alkoholtrinken andere Menschen stört oder beeinträchtigt. Dies gehört zu den für Sie weniger angenehmen Seiten des Alkoholtrinkens.

▶ *Seien Sie offen für Informationen.*

Verglichen mit Anderen nehmen Sie Informationen über mögliche negative Seiten des Alkoholtrinkens ernst. Das kann Sie voranbringen und Ihnen helfen, Vor- und Nachteile gegeneinander abzuwiegen.

▶ *Besinnen Sie sich auf Ihre Prinzipien.*

An einigen Punkten stimmt für Sie das Alkoholtrinken nicht mit Ihren Prinzipien überein. Das mag ein Argument dafür sein, weiter über Vorteile eines risikoarmen Konsums nachzudenken.

8 Bleiben Sie am Ball!

Sie haben sich jetzt recht detailliert mit dem Thema Alkohol beschäftigt. Wir hoffen, Ihnen interessante Hinweise gegeben zu haben, die Sie für Ihre Gesundheit und Ihr Wohlbefinden nutzen können. Vielleicht haben Sie beim Lesen darüber nachgedacht, wie es wohl wäre, risikoarm Alkohol zu trinken. Bleiben Sie neugierig und beobachten Sie Ihre Einstellung. Bewahren Sie den Brief so auf, dass Sie ihn vielleicht in einigen Wochen noch einmal lesen können. Oft ergeben sich beim zweiten Lesen neue Perspektiven.

PAL Pilot

Universitätsmedizin GREIFSWALD

Universitätsmedizin Greifswald / Institut für Medizinische Psychologie

Herr
Bert Beispiel
1 Beispielweg 1
11111 Beispielstadt

15. Dezember 2022

Ihre neue Rückmeldung - "Ein Weg gesund zu leben"

Sehr geehrter Herr Beispiel,

2 vielen Dank, dass Sie an einer weiteren Befragung zu Ihrem Lebensstil teilgenommen haben. Sie erhalten nun eine neue persönliche Rückmeldung zu den Themen körperliche Aktivität, Ernährung, Alkoholkonsum und Tabakrauchen. Wir teilen Ihnen Veränderungen seit der letzten Befragung mit.

Wo stehen Sie im Vergleich zu den Empfehlungen?

3

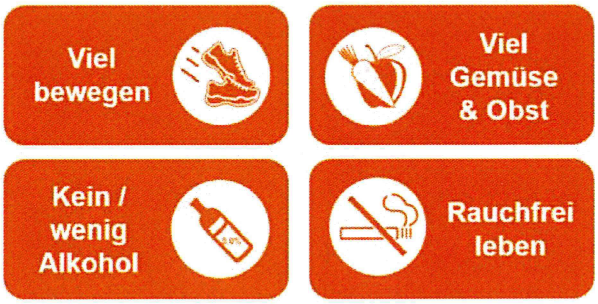

Die orangen Bilder zeigen Ihnen die Bereiche, in denen Sie aktuell die Möglichkeit haben noch etwas mehr für Ihre Gesundheit zu tun. Die orangen Symbole zeigen Ihnen, dass Sie durch mehr körperliche Aktivität, mehr Gemüse- und Obstkonsum, weniger Alkoholkonsum und/ oder einen Rauchstopp die Möglichkeit haben etwas mehr für Ihre Gesundheit zu tun. Wie beim letzten Mal, finden Sie im Folgenden eine ausführlichere Rückmeldung zu den Themenbereichen körperliche Aktivität und Rauchen.

4 Wir wünschen Ihnen, sehr geehrter Herr Beispiel, alles Gute und viel Erfolg!

Mit freundlichen Grüßen

Ihre Professorin Dr. J. Freyer-Adam

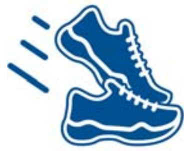

1 **Falls es Sie interessiert, erhalten Sie nun Ihre neue persönliche Rückmeldung zu Ihrer körperlichen Aktivität.**

2 **Wo stehen Sie?**

Sie sind nach wie vor zwiegespalten in Bezug auf Ihre körperliche Aktivität. Zum einen machen Sie sich Gedanken und zum anderen sind Sie nicht ausreichend überzeugt, dass Sie daran etwas ändern möchten. Vielleicht kann Ihnen Ihre neue persönliche Rückmeldung helfen weiter voranzukommen.

So hat sich Ihre körperliche Aktivität seit der letzten Befragung verändert:

1. Die Zeit, die Sie in der Woche mit körperlicher Aktivität verbringen, hat sich seit der letzten Befragung nicht verändert.

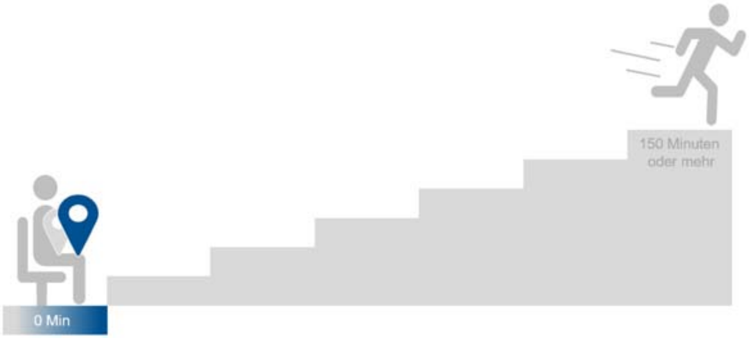

2. Sie haben nach wie vor noch keine Übungen zum Aufbau oder zur Kräftigung Ihrer Muskulatur in Ihren Wochenplan eingebaut. Vielleicht können Sie die folgenden Hinweise dazu motivieren.

3 **Erinnern Sie sich?**

JEDE körperliche Aktivität zählt!

Die Weltgesundheitsorganisation empfiehlt Menschen mit Vorerkrankungen körperliche Aktivität an ihre Möglichkeiten anzupassen und wöchentlich mindestens:

- **150 Minuten** moderate Aktivität oder 75 Minuten anstrengende Aktivität oder eine Kombination aus beidem
- und an 2 Tagen Übungen zur Kräftigung der Muskulatur anzustreben.

Es bietet sich an mit kleinen Übungen anzufangen und sich mit der Zeit zu steigern.

Moderate Aktivität:

Sie atmen und schwitzen dabei **etwas stärker** und/ oder Ihr Herz schlägt **etwas schneller** als normal. Auch das Sprechen fällt etwas schwerer.

Beispiele: schnelles Gehen, zügiges Radfahren, normales Schwimmen, Rasen mähen, Fenster putzen, leichte Lasten tragen

Anstrengende Aktivität:

Sie atmen und schwitzen dabei **viel stärker** und/ oder Ihr Herz schlägt **viel schneller** als normal. Beim Sprechen schnappen Sie nach Luft.

Beispiele: schnelles Joggen, Rennrad fahren, schnelles Schwimmen, Aerobic, Fußball spielen, Garten umgraben

4 **Sie empfinden nicht alles an körperlicher Aktivität als unangenehm.**

Sie erleben in gleichem Maße auch angenehme Seiten. Seit der letzten Befragung haben die angenehmen Seiten von körperlicher Aktivität im Vergleich zu den unangenehmen für Sie an Bedeutung gewonnen. Das ist ein wichtiger Schritt für eine erfolgreiche Veränderung!

Körperliche Aktivität hat für Sie keine oder nur wenige angenehme Seiten, wie zum Beispiel Ihre Stimmung zu verbessern. Körperliche Aktivität hat für Sie keine oder nur wenige unangenehme Seiten.

Im Vergleich zu anderen Menschen …

… finden Sie die angenehmen Seiten nicht so bedeutsam. Lassen Sie sich einmal folgende Überlegungen in Ruhe durch den Kopf gehen:

- Wenn Sie vermehrt körperlich aktiv sind, tun Sie Ihrem Körper langfristig etwas Gutes.
- Wie sieht Ihre Pro- und Kontra-Liste zu körperlicher Aktivität aus?

Oft ist es interessant zu überlegen, welche der angenehmen oder unangenehmen Seiten an körperlicher Aktivität Ihnen momentan am wichtigsten sind. Welche könnten Ihnen langfristig mehr Vorteile bringen?

5 Und wie kommen Sie weiter?

Die folgenden Empfehlungen haben sich in vielen Fällen bewährt und können Sie unterstützen, regelmäßig körperlich aktiv zu sein. Ob auch für Sie geeignete Anregungen dabei sind, können Sie für sich entscheiden.

▶*Seien Sie offen für Informationen.*

Verglichen mit anderen nehmen Sie Informationen über körperliche Aktivität nach wie vor eher weniger wahr. Viele Menschen interessieren sich nicht so sehr für Informationen zu regelmäßiger Bewegung und ihren positiven Auswirkungen. Vielleicht können Ihnen Informationen und Berichte von körperlich aktiven Personen einige Anregungen geben.

▶*Denken Sie an Nachteile mangelnder körperlicher Aktivität.*

Sie sind nach wie vor selten über Menschen verärgert, die keinen Sport machen, obwohl es ihrer Gesundheit guttun würde. Wie sieht es mit Ihnen selbst aus? Welche negativen Auswirkungen könnte zu wenig Bewegung für Sie bedeuten?

▶*Achten Sie auf negative Auswirkungen auf Ihr Umfeld.*

Sie denken heute eher weniger darüber nach, welche Auswirkungen mangelnde körperliche Aktivität Ihrerseits auf Ihr Umfeld haben kann. Vielleicht gab es für Sie dazu noch keinen Anlass. Versetzen Sie sich einmal in Ihnen nahestehende Menschen hinein: hat sich schon mal jemand besorgt über Ihre mangelnde körperliche Aktivität geäußert?

▶*Schauen Sie auf förderliche Veränderungen in Ihrer Umgebung.*

Verglichen mit anderen nehmen Sie nach wie vor weniger wahr, dass körperliche Aktivität in Ihrer Umgebung und in der Gesellschaft allgemein ein wichtiges Thema geworden ist. Vielleicht achten Sie in der nächsten Zeit vermehrt darauf, welche Berichte es zu körperlicher Aktivität gibt und was Sie in Ihrem Umfeld darüber erfahren.

6 Achtung: Zu überwindende Situationen

Wenn etwas dazwischenkommt, fällt es einem oft nicht leicht, trotzdem körperlich aktiv zu sein. Gewohnheiten zu ändern, ist dann besonders schwer. Es ist daher wichtig, sich vorher zu überlegen, wie man in solchen Situationen angemessen reagieren kann.

Sie haben nach wie vor vergleichsweise wenig Zutrauen in Ihre Fähigkeit, auch in schwierigen Situationen körperlich aktiv zu sein. Dies hindert Sie möglicherweise daran, konkrete Veränderungen zu planen. Überlegen Sie einmal: Waren Sie in der Vergangenheit schonmal vermehrt körperlich aktiv? Haben Sie schonmal andere Ziele, nach Überwindung Ihres inneren Schweinehunds, erreicht? Welche Ihrer Stärken haben Ihnen dabei geholfen und könnten auch diesmal hilfreich sein?

Ihnen fällt es besonders schwer körperlich aktiv zu sein, wenn das Wetter schlecht ist; Sie Stress haben und Ihnen kein Ort zugänglich ist, an dem Sie in Bewegung sein können. Folgendes kann hilfreich sein:

- Sich einen Plan B für schlechtes Wetter überlegen, z.B. Ersatztraining zu Hause oder im Fitnessstudio
- Statt die Sporteinheit ausfallen zu lassen, eine Trainingseinheit am nächsten Tag festlegen
- Sich mit entsprechender Kleidung ausrüsten
- Auf Online-Trainingsangebote zurückgreifen, die Sie auch von zu Hause aus angehen können
- Schauen, ob durch körperliche Aktivität der Stress nicht sogar verringert wird
- Leichtere körperliche Aktivität wie Spaziergänge, kleinere Fahrradtouren oder Yoga wählen
- Statt die Sporteinheit ausfallen zu lassen, nur die Hälfte der Zeit als Ziel festlegen
- Bewegung in den Alltag integrieren (Fahrradfahren statt Autofahren, Treppen steigen statt Fahrstuhl fahren, etwas weiter weg parken)
- Körperliche Aktivität auswählen, für die Sie keine Sportstätte brauchen
- Nach Sportkursen erkundigen, die unter freiem Himmel stattfinden

8 Bleiben Sie am Ball!

Auf dem Weg zu mehr körperlicher Aktivität haben Sie bereits eine entscheidende Hürde genommen: Sie denken darüber nach, mehr körperliche Aktivität in ihren Alltag zu integrieren. Wir hoffen, Ihnen interessante Hinweise gegeben zu haben, die Sie für Ihre Gesundheit und Ihr Wohlbefinden nutzen können. Bewahren Sie den Brief so auf, dass Sie ihn in der nächsten Zeit noch einmal lesen können. Vielleicht finden Sie dadurch zusätzlichen Schwung, um sich auf Ihr Ziel konkret vorzubereiten.

1 **Falls es Sie interessiert, erhalten Sie nun**
Ihre neue persönliche Rückmeldung zu Ihrem Rauchverhalten.

2 Wo stehen Sie?

Der Weg zum Ziel Nichtrauchen führt über fünf Etappen. Ihren Stand können Sie der folgenden Wegbeschreibung entnehmen:

1. Etappe: Absichtslosigkeit	"Ich rauche und habe nicht die Absicht, damit in nächster Zeit aufzuhören."
**2. Etappe: Absichtsbildung**	**"Ich möchte in den nächsten sechs Monaten versuchen, das Rauchen aufzugeben."**
3. Etappe: Vorbereitung	"Ich will in den nächsten vier Wochen mit dem Aufhören Ernst machen."
4. Etappe: Handlung	"Ich habe vor weniger als einem halben Jahr das Rauchen aufgegeben."
5. Etappe: Aufrechterhaltung	"Ich rauche seit mehr als einem halben Jahr nicht mehr und möchte nicht rückfällig werden."

Damit befinden Sie sich zwar noch auf der gleichen Etappe wie vor einigen Monaten, aber Sie beschäftigen sich nach wie vor mit dem Thema. Das bedeutet, dass Ihnen die Nachteile des Rauchens alles andere als gleichgültig sind. Nehmen Sie sich die Zeit, sich noch einmal damit zu befassen. Bald sind Sie vielleicht dazu bereit, das Aufhören ganz konkret zu planen! Wir helfen Ihnen weiterhin gerne dabei.

3 Rauchen oder nicht rauchen?

Sie können Ihr Wohlbefinden steigern, auch wenn Sie über 60 Jahre alt sind, indem Sie den Rauchstopp jetzt wagen. Herz und Kreislauf, Atemwege und Lunge werden entlastet und funktionieren besser. Dadurch verspüren Sie größere Lust auf Bewegung. Die Folge ist allgemein größeres Wohlbefinden und eine höhere Lebenserwartung bei guter Gesundheit.

Sie sind sich der Nachteile des Rauchens noch zu wenig bewusst. Daher möchten wir Ihnen empfehlen, sich noch weiter mit den Nachteilen des Rauchens zu beschäftigen. **Wichtig:** Das Rauchen schadet nicht nur Ihnen. Ihre gesamte Umgebung raucht mit, seien es die Kinder, die Partnerin, der Partner, Mitarbeiterinnen oder Mitarbeiter. Passivrauchende haben häufiger Atemwegserkrankungen und erleiden zum Beispiel häufiger einen Herzinfarkt als Menschen in rauchfreier Luft.

4 Achtung: Kritische Situationen

Jede Raucherin und jeder Raucher greift in ganz bestimmten Situationen besonders gerne zur Zigarette. Wie gut kennen Sie Ihre eigenen kritischen Momente, in denen es schwer fällt, zu widerstehen? Da Sie beabsichtigen, in nächster Zeit aufzuhören, sollten Sie diese Situationen kennen, damit Sie richtig darauf reagieren und trotz der Versuchung, auf das Rauchen verzichten können.

Sie verspüren oft starke Lust auf eine Zigarette. Wenn Sie das Rauchen aufgeben wollen, sollten Sie Ihre Widerstandskraft gegen kritische Situationen noch etwas verstärken:

- Überlegen Sie sich jedes Mal vor dem Anzünden einer Zigarette, warum Sie gerade jetzt rauchen wollen. Zünden Sie sie erst an, wenn Sie sich darüber im Klaren sind.
- Versuchen Sie schon jetzt, jene Zigaretten auszulassen, auf die Sie am ehesten verzichten können.

Ihnen scheinen besonders Situationen mit *anderen Rauchern* Schwierigkeiten zu bereiten. Offenbar rauchen Sie besonders gern, wenn Sie in guter Stimmung oder in Gesellschaft von anderen Raucherinnen und Rauchern sind. Überlegen Sie sich deshalb gut, wie Sie diese Situationen meistern wollen. Versuchen Sie, sich vermehrt unter Nichtraucher zu mischen. Gewöhnen Sie sich schon jetzt den Satz an: "Nein danke, ich rauche nicht mehr!".

5 Und wie kommen Sie weiter?

Die folgenden Maßnahmen können Ihnen den Ausstieg aus dem Rauchen erleichtern. Diese Empfehlungen haben sich in vielen Fällen bewährt. Aufgrund Ihrer Angaben in der Befragung können wir Ihnen ein paar maßgeschneiderte Hinweise geben, die dazu beitragen können, dass Sie Ihrem Ziel näher kommen.

▶*Vielleicht sehen Sie es ähnlich...*

Wer die Tatsache voll und ganz akzeptiert, dass das Rauchen mit schwerwiegenden Gesundheitsrisiken verbunden ist, kann das Rauchen besser aufgeben.

Möglicherweise könnten Sie folgende Tipps ein Stück weiterbringen:

- Falls Sie Fragen rund um das Thema Rauchen haben: Sprechen Sie doch Ihren Hausarzt/Hausärztin an, wenn Sie das nächste Mal die Sprechstunde aufsuchen. Sie erhalten auch hier weitere wichtige Informationen.
- Achten Sie in den kommenden Wochen bewusst auf alle Informationen über die Schädlichkeit des Rauchens in den Medien.

▶*Fassen Sie einen klaren Entschluss*

Wenn Sie daran glauben, dass Sie ohne Zigaretten vieles in Ihrem Leben positiver gestalten können, fällt Ihnen das Aufhören bedeutend leichter.

Was hindert Sie daran, all die Vorteile des Nichtrauchens in Betracht zuziehen? Dazu einmal drei Gedanken an dieser Stelle:

- Listen Sie auf einem Blatt Papier die Gründe auf, warum Sie noch rauchen. Zum Beispiel: "Weil mich Rauchen entspannt", "Weil ich mich so besser konzentrieren kann". Dann erstellen Sie eine zweite Liste mit Gründen, die für ein baldiges Aufhören sprechen. Zum Beispiel: "Ich werde ein Gesundheitsrisiko los", "Meine Kleider riechen nicht mehr nach Rauch". Welche der beiden Listen ist stichhaltiger?
- Die besten Auskünfte über die Vorteile des Aufhörens erhalten Sie von Ex-Rauchenden. Kennen Sie in Ihrem Umfeld solche Menschen?
- Seit einigen Jahren haben sich die Einstellungen zum Rauchen stark verändert. Es werden zunehmend Nichtraucherzonen eingerichtet. Immer mehr Menschen geben das Rauchen auf. Ist der Trend Nichtrauchen möglicherweise auch eine Chance für Sie?

▶*Rauchen Sie bewusster*

Wenn Sie Kontrolle über Ihre Rauchgewohnheiten gewinnen, d.h. wenn Ihnen bewusst wird, wann und warum Sie besonders gerne rauchen, können Sie bald in vielen Situationen leichter auch ohne eine Zigarette auskommen.

Seine eigenen Rauchgewohnheiten zu kennen, kann hilfreich sein. Vielen Menschen hilft es, zu überlegen, warum sie die Zigarette rauchen wollen, bevor sie sie anzünden. So wird es leichter, auf die eine oder andere Zigarette zu verzichten. Versuchen Sie es doch einmal so:

- Zünden Sie jene Zigaretten nicht sofort an, auf die Sie am ehesten verzichten können.
- Versuchen Sie an bestimmten Orten, z.B. in Ihrer Wohnung, am Arbeitsplatz oder im Auto, auf das Rauchen zu verzichten.
- Kaufen Sie nur noch einzelne Päckchen. Je geringer Ihr Zigarettenvorrat ist, desto geringer ist auch die Versuchung zu rauchen.

▶*Lassen Sie sich unterstützen*

Es ist immer nützlich, mit verständnisvollen Menschen reden zu können. Das gilt auch dann, wenn es darum geht, Ihren Vorsatz, das Rauchen aufzugeben, in die Tat umzusetzen. Geben Sie den Menschen in Ihrer Umgebung Ihren Entschluss bekannt. Lassen Sie sich von Ihren Angehörigen und Freunden ermutigen!

Wir empfehlen Ihnen, vermehrt solche Kontakte zu suchen: in der Familie, bei Freunden und Freundinnen. Vor allem bewährt hat sich das Gespräch mit Menschen, die selber das Rauchen aufgegeben haben.

▶*Lenken Sie sich vom Rauchen ab*

Der Versuchung zu rauchen kann man sich mit verschiedenen Mitteln entziehen. Sie wissen ja längst, dass nicht jede Zigarette "nötig" ist. Vielleicht denken Sie, wenn Sie sich Ihre nächste Zigarette anzünden, darüber nach, was Sie stattdessen tun könnten.

Versuchen Sie, öfter Tricks wie die folgenden anzuwenden.

- Trinken Sie ein Glas Wasser oder essen Sie einen Apfel.
- Nehmen Sie einen Kaugummi oder ein Bonbon in den Mund.
- Atmen Sie tief durch oder gehen Sie für ein paar Schritte an die frische Luft.
- Warten Sie fünf Minuten ab, bis das Verlangen nach einer Zigarette abklingt.

So können Sie Erfahrungen sammeln, wie Sie der Lust auf eine Zigarette am besten entgehen.

6 Bleiben Sie am Ball!

Auf dem Weg zum Nichtrauchen haben Sie eine entscheidende Hürde überwunden. Sie haben sich vorgenommen, mit dem Rauchen aufzuhören. An diesem Punkt hat es anderen Menschen geholfen, genau zu planen, welche konkreten Schritte man nun unternehmen könnte, um mit dem Rauchen aufzuhören. Haben Sie schon Ideen dazu?
