# Supplementary material for: General hospital patients’ satisfaction with a proactive automatized multiple health behavior change intervention
Source: BMC Health Serv Res. 2025 Sep 23;25:1209. doi: 10.1186/s12913-025-13425-x (PMC12459032; doi:10.1186/s12913-025-13425-x)
Supplement: Supplementary file 2 — Supplementary Material 2 [file 12913_2025_13425_MOESM2_ESM.pdf]

Additional file 2: Multi-dimensional treatment satisfaction measure

Domain	Attribute	Subscale	Item
**Process**	Intervention components	Suitability	1. Feedback was comprehensible<br>2. Feedback was tailored to my personal situation<br>3. Feedback was presented attractively<br>(0, not at all – 4, very much)
		Utility	1. Feedback was helpful<br>2. Feedback was motivating<br>3. Feedback was interesting<br>4. Feedback was respectful<br>(0, not at all – 4, very much)
	Overall intervention	Attitude toward intervention	1. Expectations were fulfilled<br>2. Participation was worthwhile<br>(0, not at all – 4, very much)
		Recommendation	1. Repetition of participation<br>(0, not at all – 4, very much)<br>2. Recommendation to other persons<br>(0, not at all – 4, very much)
	Implementation	Format and dose	1. Amount per feedback was appropriate<br>2. Frequency of feedback was appropriate<br>3. Intervals between feedbacks were appropriate<br>(0, not at all – 4, very much)<br><br>If scores <3:<br>1a. What amount of feedback would you consider as appropriate? (fewer / more)<br>2a. What frequency of feedback would you consider as appropriate? (fewer / more)<br>3a. What intervals between feedbacks would you consider as appropriate? (smaller / larger)
**Outcome**	Attribution of intentional changes in HRBs to intervention		1. Intervention encouraged me to make changes<br>(0, not at all – 4, very much)
	Perceived benefits		Because of the intervention I intended to …<br>…1. smoke less (or no) tobacco<br>...2. drink less (or no) alcohol<br>…3. eat healthier<br>…4. be physically more active<br>(yes / no)
	Discomfort		Experienced discomfort or unpleasant side effects<br>(yes/ no)<br>If yes:<br>Please describe discomfort
